# Vestibular paroxysmia: a systematic review

**DOI:** 10.1007/s00415-025-12913-8

**Published:** 2025-02-11

**Authors:** Marianne Dieterich, Thomas Brandt

**Affiliations:** 1 German Center for Vertigo and Balance Disorders, University Hospital, Ludwig-Maximilians University, Marchioninistrasse 15, 81377 Munich, Germany; 2 Department of Neurology, LMU University Hospital, Munich, Germany

**Keywords:** Vertigo attacks, Audiovestibular deficits, Neurovascular cross compression, Vestibular excitation, Sodium channel blockers

## Abstract

The key symptoms of vestibular paroxysmia (VP) due to neurovascular cross-compression (classical VP) or compression of the eighth nerve by space-occupying cerebellar-pontine angle processes (secondary VP) are frequent short attacks of vertigo and dizziness with unsteadiness which last seconds to minutes. They can be accompanied by unilateral auditory symptoms such as tinnitus or hyperacusis. Head movements and hyperventilation can induce nystagmus and VP attacks that most often occur spontaneously. VP is diagnosed in 3% of patients in a tertiary vertigo care center and very rarely affects children. The mean age of first appearance is 47 to 51 years with equal sex distribution. A combination of high-resolution MRI sequences (with constructive interference in steady-state/fast imaging employing steady-state, 3D-CISS/ FIESTA) of the cerebello-pontine may support the diagnosis although the beneficial treatment with sodium channel blockers is the most reliable clinical sign for classical VP, secondary VP and idiopathic VP (without verification of a causative pathology). Because of the frequency, shortness, and audiovestibular symptomatology of the attacks, the differential diagnosis to other conditions such as paroxysmal brainstem attacks, vestibular epilepsy, rotational vertebral artery compression syndrome or “near”-narrowed internal auditory canal syndrome is only relevant in exceptional cases. However, imaging of the posterior fossa including the inner ear is mandatory to distinguish between classical, secondary and idiopathic VP forms. Randomized controlled trials for medical treatment are still needed. Practical therapy of choice is medical treatment with sodium channel blockers (carbamazepine, oxcarbazepine, lacosamide). A microsurgical decompression is effective in secondary VP but is the ultimate therapy in cases with classical or idiopathic VP when medication is not tolerated.

## Introduction

Vestibular paroxysmia (VP) is a clinically highly relevant, rare vertigo syndrome with well-defined diagnostic criteria and an effective medical treatment. The main symptoms are recurrent short attacks of rotatory or swaying vertigo with unsteadiness of stance or gait which last seconds to minutes and occur with or without unilateral ear symptoms such as tinnitus or hypoacusis. The frequent attacks happen spontaneously and can be elicited in certain patients by head positions or movements. Sometimes attacks are induced by hyperventilation. Mild symptoms such as tinnitus or hypoacusis can also be present during the attack-free intervals. The goal of this review was to study (PubMed literature search) and present the current literature on VP and the most adequate imaging technique.

The first description of short “hyperactive dysfunction symptoms of the eighth cranial nerve” was made in 1975 by Jannetta [1] who attributed it to a neurovascular cross-compression and later named it “disabling positional vertigo” in 1986 [2]. The clinical characterization was heterogeneous with a broad variability of features (spinning vertigo, light-headedness, instability of stance and gait without vertigo), attack duration (from seconds and minutes to days), and accompanying symptoms [3, 4]. Thus, this clinical manifestation made it indistinguishable from other episodic vertigo syndromes such as vestibular migraine, superior canal dehiscence syndrome or functional dizziness. The term “vestibular paroxysmia” (VP) was first introduced by Brandt and Dieterich in 1994 who specified the syndrome including the beneficial medical treatment with the antiepileptic drug carbamazepine [5]:

- Short attacks of spinning or non-spinning vertigo for seconds to minutes
- Attacks frequently dependent on a particular head position
- Hyperacusis or tinnitus during the attacks or constantly present
- Neurophysiological evidence of a vestibular or auditory deficit
- Efficiency of carbamazepine

For diagnosis of this observational study, at least three of the first four criteria together with the beneficial response to the sodium channel blocker carbamazepine were required [5, 6].

In a follow-up study of a larger cohort of patients, the diagnostic criteria for VP distinguished between definite VP and probable VP based on more detailed signs and symptoms, provoking factors and neurophysiological findings [7].

In 2016, the Classification Committee of the international Bárány Society formulated the modified diagnostic criteria for VP in its current version (Table 1) [8]:

**Table 1:** Diagnostic criteria for VP

**Definite vestibular paroxysmia** (each point needs to be fulfilled)
1. At least 10 attacks of spontaneous spinning or non-spinning vertigo
2. Duration less than 1 min
3. Stereotyped phenomenology in a particular patient
4. Response to treatment with a sodium channel blocker
5. Not better accounted for by another diagnosis.
**Probable vestibular paroxysmia** (each point needs to be fulfilled)
1. At least 5 attacks of spinning or non-spinning vertigo
2. Duration less than 5 min
3. Spontaneous occurrence or provoked by certain head movements
4. Stereotyped phenomenology in a particular patient
5. Not better accounted for by another diagnosis.

In analogy to trigeminal neuralgia [9], we recommend three clinical forms of VP: Classical VP by neurovascular cross-compression with morphological proof of a proximal damage of the eighth nerve; secondary VP due to other space-occupying etiologies such as tumors, cysts, narrow internal auditory canal syndrome or pathological arteries; and idiopathic cases without verification of a causative pathology. What they all have in common is the clinical manifestation of frequent short dizziness attacks and the response to medical treatment with sodium channel blockers.

### Patient history

The key symptom of VP is frequent recurrent short attacks of spinning or non-spinning vertigo, which typically last for seconds to a minute, but occasionally longer. In extreme cases, up to 70 attacks per day can occur. In the vast majority of cases, the attacks occur spontaneously. In some patients, attacks can be induced, for instance, by head turns to the right or to the left or other head movements. In these cases, one should be careful to exclude the different forms of benign paroxysmal positional vertigo (BPPV) in which the typical attacks and nystagmus are elicited by canalolithiasis when head movements are performed in the plane of a particular semicircular canal relative to the gravitational vector. In VP, vertigo and nystagmus can also be induced by hyperventilation [7, 10].

In a study with 146 patients (73 definite VP, 73 probable VP), the frequency of the attacks ranged between 5 and 30 attacks per day [11]. Spinning vertigo was the most frequent type; this was more frequent in patients with definite VP than in those with probable VP. In two-third of patients, attacks occurred spontaneously; in one-quarter, they were triggered by head movements. The majority (approximately 70%) did not report any accompanying symptoms; in about 20–30%, mild unilateral cochlear symptoms prevailed [11]. In another study, one-third of patients initially showed hyperventilation-induced nystagmus and in some patients, the attacks were associated with hyperacusis or hypoacusis and/or tinnitus in the affected ear [12]. Nausea, vomiting, loss of consciousness and falls are not typical features of the condition.

As mentioned above, a convincing response to treatment with sodium channel blockers, such as oxcarbazepine, carbamazepine, or lacosamide (see below), in adequate dosages is important for the definite diagnosis. As long as the effect of the treatment with sodium channel blockers is not known, only the diagnosis of probable VP can be made (see differential diagnosis) [8].

There are also patients who have, in addition to the recurrent attacks of vertigo, other neurological symptoms and signs, e.g., facial hemispasm [13–15], which is due to a combined irritation of the cranial nerves VIII and VII in the meatus acusticus internus where both nerves lie close to each other. The combination with trigeminal neuralgia and facial hemispasm, another two types of neurovascular compression of a cranial nerve, was also described [16]. There is an analogous disease due to a compression of the cochlear nerve characterized by recurrent brief attacks of tinnitus (so-called typewriter tinnitus)[12, 17, 18]; here again the diagnosis can only be made based on the beneficial effect of sodium channel blockers.

### Epidemiology

**Fig. 1:**
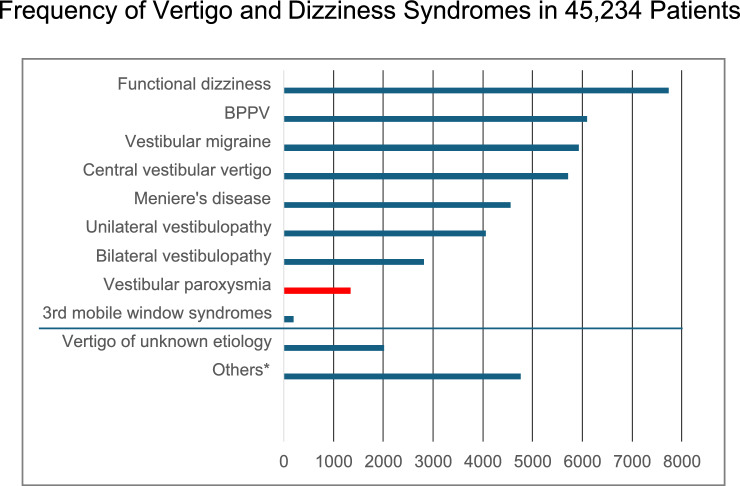
Frequency of different vertigo and dizziness syndromes in 45,234 patients from a tertiary interdisciplinary vertigo outpatient unit of the German Center for Vertigo and Balance Disorders and the Department of Neurology, LMU University Hospital, Munich, Germany, over 26 years from 1998 to 2023. VP is a rare vestibular disease accounting for 3% (*N* = 1339) of vertigo/dizziness patients. *Others means dizziness or imbalance due to non-vestibular origin, e.g., polyneuropathy, orthostatic dizziness, ocular motor disorders

Although there are no data on the lifetime prevalence of VP, it can be considered a rare disease. Because of the rarity of the disease, studies with large numbers of cases do not exist so far. A few case series and several single cases have been published (e.g., [5–7, 11, 19–25]). The relative frequency of VP in a group of 45,234 patients with vertigo and dizziness in a tertiary vertigo care center was 3% (Fig. 1). The mean age of the patients, for instance in the four case series with more than ten patients, was 47–51 years (range 17–78 years) [5, 7, 19, 25]. In these four studies, 45 of the total number of 81 patients were female (55.6%), i.e., there was no obvious sex preponderance.

VP was also described occasionally in children, with features similar to those in adults [22, 26].

### Clinical examination

During the attack-free interval in about 20% of patients, there is evidence of a mild unilateral peripheral vestibular hypofunction (pathological head-impulse test, head-shaking nystagmus) or a slight impairment of hearing. In some patients, hyperventilation or head-shaking can induce vertigo and nystagmus, which can change its direction [7, 10, 11]. During an attack, there is typically a spontaneous nystagmus, which can be due to an excitation or transient loss of function or change its direction if there is a transition between the two [15, 27].

### Neurophysiological examinations

During the attack-free interval**,** about 33–50% of patients show mild to moderate peripheral vestibular and/or cochlear deficits [7, 11, 19, 25]. Impairment of hearing is much less pronounced than in most cases of Menière’s disease. In addition to mild vestibular hypofunction, an excitation or a combination of excitation and inhibition in various tests (subjective visual vertical, measurement of ocular torsion, caloric testing, or vestibular-evoked myogenic potentials) can also be present [19, 25]. A study with auditory brainstem responses showed that patients with VP had longer interpeak latencies I–III and wave III latencies compared to non-VP patients, which also supports the assumed pathophysiology of a neurovascular compression [28].

During the attack, in a well-documented case with right-sided neurovascular cross-compression, initially a left-beating nystagmus was recorded by videooculography, which, after 47 s, beat to the right for 10 s. This is compatible with an inhibition and excitation of the vestibular nerve [27, 29]. In another case, an excitatory nystagmus was recorded; in this case, treatment with a sodium channel blocker was effective [30].

### Imaging VP

**Fig. 2:**
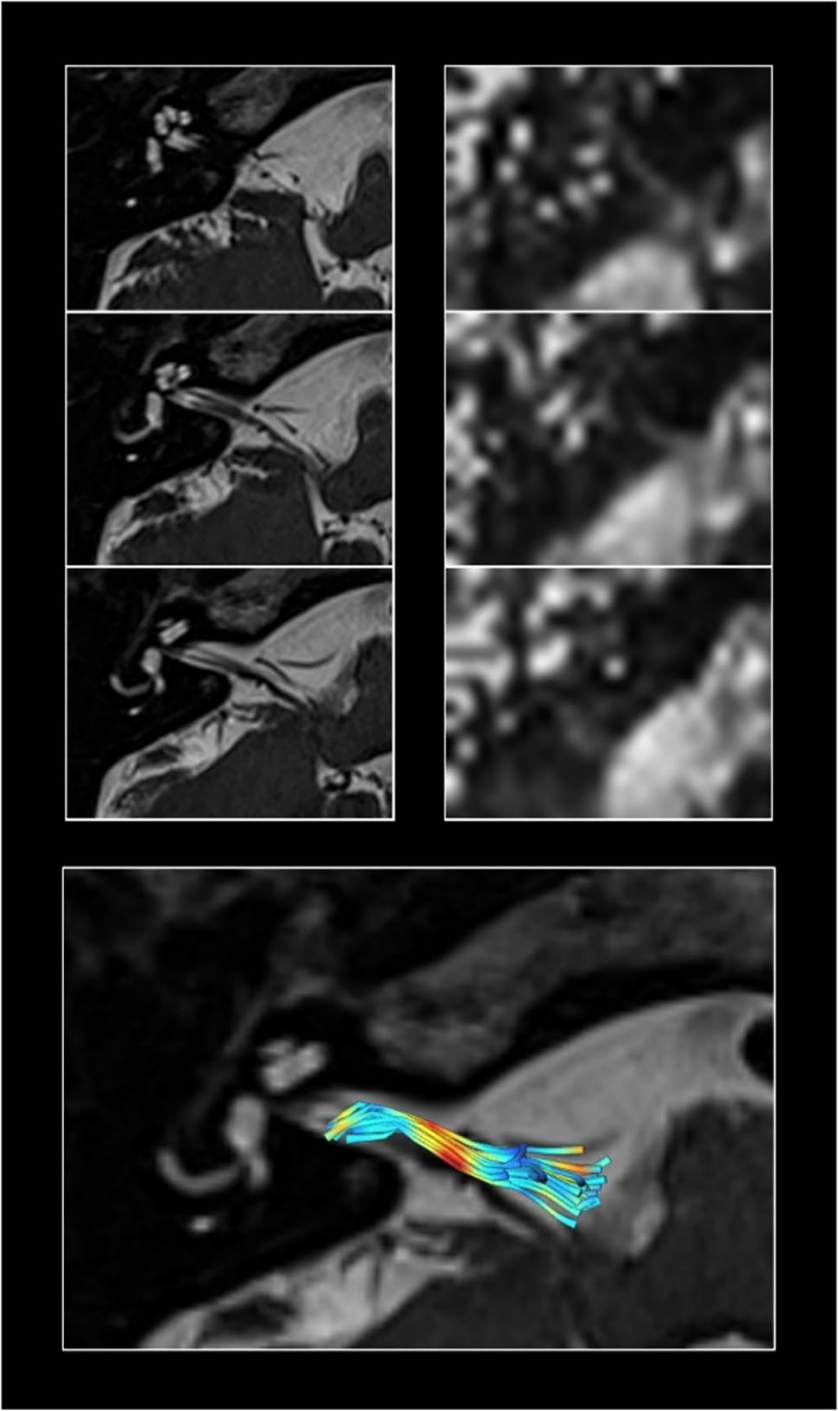
Example of the cranial MRI of a VP patient. Constructive interference in steady-state sequence (CISS) with 0.5 × 0.5 × 0.5 mm for high-resolution structural imaging (left column) depicting the semicircular canals and the vestibular nerve with the root entry zone into the pontomedullary brainstem. Reconstruction of fractional anisotropy based on 2 × 2 × 2 mm diffusion-weighted imaging (DWI)(right column).While structural MRI (left column) can reveal fine details of vestibulocochlear nerve anatomy, such as the presence and extent of neurovascular contact and associated nerve angulation, it cannot provide information on nerve integrity. For this, DWI data are used (right column) since the reconstruction of specific diffusion tensor maps from raw DWI data can relay further information on voxel-wise physical properties such as fractional anisotropy. Fiber-tracking algorithms allow to determine the three-dimensional directional course of nerve fibers. Paired with high-resolution structural data, the vestibulocochlear nerve can be identified and disease-specific changes in nerve integrity can be measured (bottom). Lower nerve integrity is shown in yellow–red colors

The role of MRI in demonstrating a neurovascular compression and identifying the affected side has been evaluated in several case series (Fig. 2). In a study of 32 patients with VP, neurovascular compression of the eighth cranial nerve was detected in 95% and with bilateral neurovascular compression in 42% of the patients by a high-resolution MRI with constructive interference in steady-state/fast imaging employing steady-state (3D-CISS/ FIESTA) sequences of the brainstem [7]. In another study of 20 VP patients, neurovascular compression of the eighth cranial nerve was found in all patients, but also in 7 out of 20 control subjects [19]. Combining magnetic resonance imaging (MRI) sequences that permit the determination of vestibular nerve angulation (i.e., change of nerve caliber or direction), structural nerve integrity via diffusion tensor imaging (DTI), and exclusion of endolymphatic hydrops (ELH) via delayed gadolinium-enhanced MRI of the inner ear could increase the diagnostic accuracy in patients with VP [25]. In the latter study on 18 VP patients and 18 healthy controls, a neurovascular compression could be documented in 15 of 18 patients and in 10 of 18 controls; the mean distance between the brainstem and the vessel was 1.5–9.6 mm [25]. In all VP studies, the distance between the brainstem and compressing vessels varied between 0.0 and 10.2 mm. The distance seems to be one important aspect since this part of the nerve is proximal to the transition zone (up to 15 mm [31]) and covered by oligodendrocytes. However, this distance was similar in the healthy controls [25].

The involved vessel can vary. In 15 of 20 VP cases, the compressing vessel was the anterior inferior cerebellar artery (AICA, 75%), the posterior inferior cerebellar artery in one, a vein in two, and the vertebral artery in another two cases [19]. In the other study on 15/18 VP patients and 10/18 controls, the contacts were also documented most often between the anterior inferior cerebellar artery (VP 67%, controls 100%), followed by the posterior inferior cerebellar artery (VP 2/15), the superior cerebellar artery (VP 1/15), the vertebral artery (VP 1/15) and a vein (VP 1/15) [25]. Nerve angulation at the contact side was found in 53% (8/15) of VP, but only in 2 of 10 controls [25]. Furthermore, DTI structural integrity was reduced on the side affected by VP, and mild endolymphatic hydrops (61.1%) and higher asymmetry indices of the endolymphatic space were further found compared to the controls. Disease duration and total number of attacks correlated with the decreased structural integrity of the affected nerve in DTI. Distance of neurovascular compression within the nerve’s root entry zone correlated with nerve function, nerve integrity loss, and volume of the endolymphatic space in VP [25]. In an earlier smaller case series, the nerve angulation was already described in VP [32].

Thus, in summary, a high-resolution MRI with 3D-CISS/ FIESTA sequences of the brainstem allows a simple depiction of a neurovascular contact [33, 34]. However, it is not suitable as a proof of a symptomatic VP since it can be seen in up to 35–55% of healthy controls. Symptomatic VP requires the typical signs and symptoms and, in addition to the neurovascular contact, several pathological imaging parameters, such as the disturbance of microstructure and ELS changes in the root entry zone of the eighth cranial nerve.

This is also the problem of a systematic review and meta-analysis on the association of vascular loops within the internal auditory canal with audio–vestibular symptoms as performed in 16 studies and 3,455 ears by Cowen et al. [35] who were critical of the selection bias, small number of eligible studies, and heterogeneity. Despite that, they found two remarkable associations between vessels and nerves, the first a neurovascular compression of the eighth nerve associated with VP and the second intrameatal vascular loops associated with sudden onset of sensorineural hearing loss [35].

## Pathophysiological Mechanisms

**Fig. 3:**
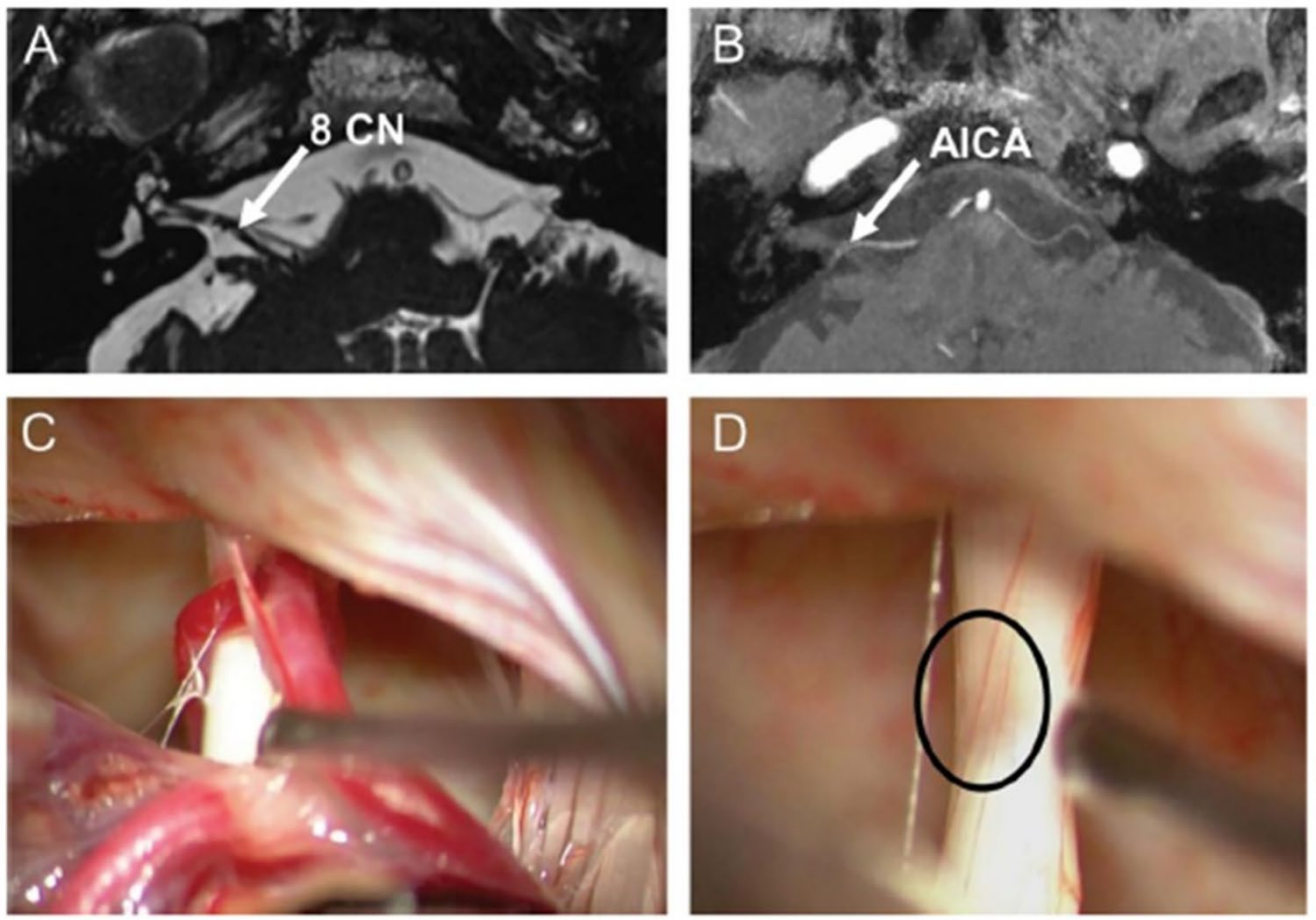
Cranial MRI of a patient with VP who had microsurgery because of intolerable medical side effects. **A** Constructive interference in steady-state sequence (CISS) and **B** time-of-flight (TOF) shows contact (arrows) between the right eighth cranial nerve (8 CN) and the anterior inferior cerebellar artery (AICA). **C** Intraoperative micrographs demonstrate vascular contact and **D** considerable compression of the eighth nerve after removal of the arteries (circle). (Modified from [23])

In 1975, Jannetta and colleagues were the first to describe the pathophysiology of VP as a “neurovascular cross-compression in patients with hyperactive dysfunction symptoms of the eighth cranial nerve”.^1^ In analogy to the brief recurrent symptoms in trigeminal neuralgia, hemifacial spasm, glossopharyngeal neuralgia, or myokymia of the superior oblique muscle [9, 36–38], it is assumed that the short attacks of vertigo are caused by compression of the vestibular nerve. Pathophysiologically, a compression can cause partial demyelination of axons, which can lead to ephaptic discharges, i.e., pathological paroxysmal inter-axonal transmissions between neighboring axons which can be further triggered by the pulsations of the artery and by change of sensory input during head movements. The hypothesis of excitation of the vestibular nerve is supported (i) by cases in which a patient becomes free of symptoms by surgical decompression [23], (ii) by nystagmus due to hyperexcitability during an attack [30], (iii) by a transition of nystagmus caused by hypo- and hyperexcitability [27, 29] and (iv) by evidence for hyperexcitability and vestibular deficits found even between the attacks [19] (Fig. 3). A 7 Tesla MRI in six patients with VP confirmed neurovascular cross-compression but did not detect structural abnormalities in these patients [39].

More detailed MRI data provide evidence that combining MRI sequences, which permit the determination of vestibular nerve angulation (i.e., change of nerve caliber or direction), structural nerve integrity via diffusion tensor imaging (DTI), and evaluation of the endolymphatic space via delayed gadolinium-enhanced MRI of the inner ear, does increase the diagnostic accuracy in VP patients [25]. The nearer the nerve–vessel contact was to the point of entry/exit of the nerve into/from the brainstem, the poorer the audiovestibular function and nerve structural integrity, and the larger the inner ear endolymphatic space were [25]. The intracisternal part of the eighth nerve starts covered by central myelin (i.e., oligodendrocytes, also called root entry zone), then 6–15 mm [31] as measured from the point of entry/exit oligodendrocytes change into Schwann cells [40], which is called the transition zone. The additional interparametric analyses corroborated the pathophysiological theory [5, 6, 19, 41] that the intracisternal part of the eighth cranial nerve covered by central myelin (i.e., oligodendrocytes) seems to be particularly vulnerable for deficits induced by the nerve–vessel contact and that the contact seems to be a necessary precondition factor for developing VP [25].

Since recurrent attacks of vertigo can also be caused by other rare pathologies (i.e., secondary VP), a cranial MRI should be always performed to document or exclude the presence of a tumor in the area of the cerebellopontine angle [42] such as vestibular schwannoma (e.g., [43]), arachnoid cysts [27], vertebrobasilar dolichoectasia [16], tortuous vertebral artery [15], or other brainstem lesions such as brainstem melanocytoma [44]. The secondary VPs were also reported to respond to the sodium channel blocker carbamazepine [15, 16].

**Fig. 4:**
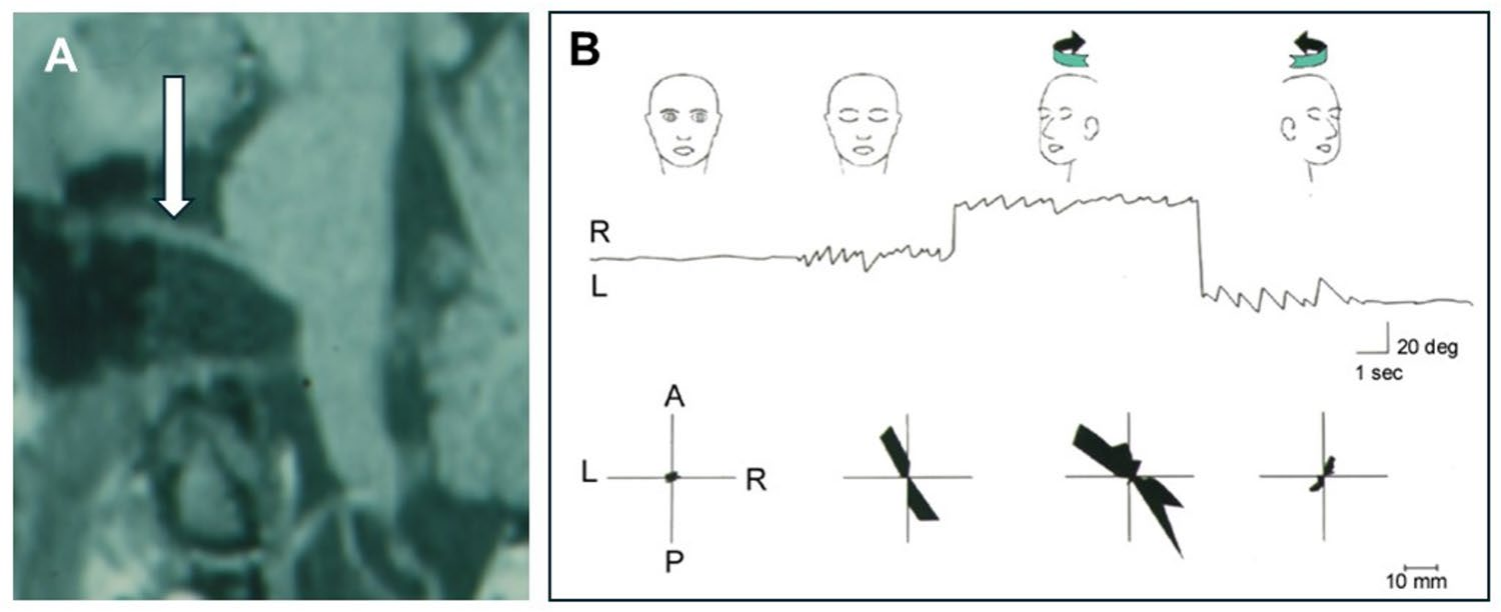
Secondary VP due to an arachnoid cyst in the right cerebellopontine angle that distorted the right vestibulocochlear nerve (**A** arrow at the nerve above the cyst in MRI scan) causing recurrent episodes of oscillopsia, rotational vertigo, and postural imbalance. These attacks were elicited and modulated by changing horizontal head positions. **B** Electronystagmography (middle) and posturography (bottom) during different head positions from straight ahead with eyes open (left) and eyes closed to head turn to the right and head turn to the left (right). Note the variation of nystagmus direction and intensity and the postural sway which was largest during head turn to the right. Ocular motor analysis revealed two different types of attacks, first, episodes of vestibular hypofunction (conduction block) for minutes to several hours, and second, **B** paroxysmal vestibular excitation (ectopic discharges) for seconds with head rotation to the right. One week after resection of the cyst and decompression of the eighth nerve the patient was symptom free and the ocular motor analysis normal. (Modified from [27])

Recurrent VP attacks induced by hyperventilation and associated with nystagmus can also occur in patients with a vestibular schwannoma [43]. Rare causes of short and head position-dependent attacks of vertigo are moreover (a) an arachnoidal cyst, which leads to an extension of the nerve [27] or (b) a cerebellopontine meningioma [30, 42] or (c) a vestibular neuritis [45]. In the case of an arachnoidal cyst, the symptoms could be explained by a conduction block of the nerve, lasting hours to days, in combination with additional head position-dependent excitation of the nerve (ectopic discharges), which led to direction-changing nystagmus for seconds to minutes (Fig. 4) [27]. Transitions from conduction block to entopic discharges have been described to occur with varying peripheral nerve compressions [46, 47].

### Therapy

Based on the assumed pathophysiology and in analogy to other neurovascular cross-compression syndromes, the first choice is a prophylactic therapy of the attacks with sodium channel blockers [5–7] because they reduce the excitability of the peripheral nerves [38]. If the diagnosis is proven with fulfillment of the current diagnostic criteria (Table 1) and a convincing response to treatment, the affected side is definitely identified and the patient does not tolerate pharmacotherapy, in rare individual cases surgical treatment [1, 48, 49] can be considered.

### Medication

There is only one randomized, placebo-controlled trial available so far [50]. The therapeutic effect of carbamazepine, which is thought to block the use-dependent fast voltage-gated sodium channels, has been described in non-controlled case series. In one of these case series, 11 out of 11 patients responded well [5]. In another, 7 out 8 patients showed an improvement [51]. In an observational follow-up study in 32 patients with a mean follow-up period of 3 years, a significant and persistent reduction of the frequency of attacks to 10% of the initial frequency and a reduction of the intensity and duration of the attacks were found during treatment with carbamazepine or oxcarbazepine [7]. In a randomized, placebo-controlled trial, a significant therapeutic effect of oxcarbazepine (900 mg/d), which has fewer side effects and interactions than carbamazepine, was found (reduction to 0.53). However, the drop-out rate due to adverse events was 60% [50].

Since there can be several contraindications, side effects and interactions for carbamazepine and oxcarbazepine, with the result that the compliance, persistence and adherence of patients can be quite low, an alternative medication is wanted. A well-tolerated [52] and evidently effective [53] alternative could be lacosamide; its primary mode of action, a blocking of sodium channels, is similar to carbamazepine but with fewer adverse effects [53].

In the very rare manifestations of VP in childhood, attacks usually cease after low-dose carbamazepine administration (2–4 mg/kg daily) [22]. Operative decompression should only be considered as a last resort since vascular, brain, and bone structures grow at different speeds in children, so that compression syndromes mostly resolve spontaneously [54].

*Pragmatic treatment* To minimize adverse effects at the beginning of the medication, a slow increase in dosing is advantageous, e.g., with 2 times 100–200 mg/d. To evaluate the efficacy of medication, e.g., oxcarbazepine (300–900 mg/d) or carbamazepine (200–800 mg/d), a treatment period of at least 4 weeks is recommended and the effects should be documented by a patient’s diary in which the number of attacks per day is noted. A positive response supports the diagnosis. In case of intolerance to these drugs, lacosamide (100–200 mg/d, up to 400 mg/d), which is well-tolerated, can be given. For other sodium channel blockers, such as phenytoin or lamotrigine, there are no case series or clinical studies yet.

### Surgical treatment

In 1986, Jannetta and colleagues reported a case series of 21 patients treated by microvascular decompression, 16 of whom benefited from this procedure [2]. These results were confirmed by larger case series on 41 patients [3] and 207 patients [4] with a success rate of between 73 and 80%. Later it was criticized that the selection criteria for surgery were variable and that no standardized outcome measures were used [48]. More recent clinical long-term observations of keyhole microvascular decompression with local anesthesia showed, in 11 of 12 VP patients, a neurovascular compression induced vertigo intraoperatively and a complete disappearance postoperatively over a mean follow-up of 116 months [55]. However, since not all patients improved with microvascular decompression and the rate of symptomatic improvement in the patients with a preoperative CISS/FIESTA MRI was greater than those without preoperative high-spatial resolution imaging [34], the authors stated that no definite conclusions could be made.

Despite the reports of partial successes, operative microvascular decompression should be reserved (as the treatment of last resort) for cases with VP with the following prerequisites: (i) the diagnosis is certain with a convincing response to pharmacotherapy; (ii) various medications are not tolerated; and (iii) the affected side is clearly identified. These restrictions have to be considered because of the risk of a brainstem and/or cerebellar infarction due to intra- or post-operative vasospasm (1–3%). Since the labyrinthine artery originates from the anterior inferior cerebellar artery (AICA) and the AICA is the compressing vessel that has to be moved in 75%, an infarction can also cause acute hearing and/or vestibular loss.

### Course of disease

Studies on the long-term course of the disease exhibited contrary results. In one study of 61 patients with a mean follow-up of 3.4 years, 44 patients (72%) still experienced vertigo attacks and 71% reported having limitations of their quality of life [24]. The authors concluded that there is a rather unfavorable prognosis in VP patients in terms of vertigo attacks and quality of life and recommended that after the initial diagnosis, follow-up is warranted to monitor clinical outcomes. In another study with 146 VP patients, three-quarters of the patients remained attack-free during a mean follow-up of 4.8 years, more than half of them even without any medication [11]. Here, the long-term prognosis of VP appears favorable, not necessarily requiring ongoing treatment. This was in line with an earlier follow-up on 32 VP patients over a mean time of 31.3 months in which treatment with carbamazepine or oxcarbamazepine led to a reduction of attack frequency and duration to 10% of baseline [7]. Thus, regular follow-up examinations are necessary to evaluate the course of the disease as well as the effects and side effects of the medical treatment to adjust the dosage.

### Differential diagnosis

To make the diagnosis, it is important that the recurrent spontaneous short attacks of vertigo respond to treatment with a sodium channel blocker and that other pathologies are excluded by MRI. There are several other disorders which may present with the leading symptom of recurrent spontaneous short attacks of vertigo. The most important differential diagnoses are short vertigo attacks due to brainstem plaques in multiple sclerosis or brainstem infarctions which both can lead to paroxysmal brainstem attacks presenting with vertigo, dysarthria, or ataxia. They may be difficult to distinguish as they can also lead to ephaptic discharges of neighboring fibers of the brainstem paths and can also respond to low doses of sodium channel blockers [56]. They are easier to diagnose when dysarthria and ataxia are more prominent and induced by changing body position (when standing up). In such cases, the use of MRI with thin brainstem slices is useful for establishing the diagnosis.

Another rare cause is an epilepsy with vestibular aura (vestibular epilepsy) that can manifest with short attacks of vertigo often lasting a few seconds [57] and nystagmus [58] primarily associated with temporal lobe seizures. Vestibular aura with additional symptoms, so-called non-isolated vestibular aura, is much more prevalent than isolated vestibular aura. A retrospective analysis of 31 patients who were diagnosed as having vestibular epilepsy, some features of which were triggered by peripheral vestibular stimuli [59], may suffer from the diagnostic uncertainty of differentiating epileptic seizures elicited by cortical excitation from peripheral nerve vestibular paroxysmia. Although the authors suggested the possibility of a single nosological entity [59], it is more likely that they are different entities and should not be confused as a common disorder as was published for “idiopathic vestibular epilepsy in dogs” [60].

Short attacks of vertigo typically provoked by head turns can exceptionally be due to a rotational vertebral artery compression syndrome that transiently reduces blood flow to the brainstem and labyrinth. These attacks may also be associated with a nystagmus indicating an excitation [61, 62].

The rare “near”-narrowed internal auditory canal syndrome can mimic VP in adults [63] and children [26] presenting with positional vertigo and exercise- or rapid head movements-induced vertigo and dizziness. The narrowed internal auditory canal corresponds to a diameter of less than 2 mm on CT or 2.9 mm on MRI in coronal sections, usually combined with a hypoplastic nerve. Similar clinical presentations were reported in a few case reports of osteoma or exostosis of the internal auditory canal [64–66].

In orthostatic hypotension, the leading symptom is recurrent attacks of dizziness or light-headedness, not vertigo, which are induced by changes of body position from lying or sitting down to an upright position [67].

Benign paroxysmal positional vertigo (BPPV) is not an often-needed differential diagnosis because it is accompanied by a crescendo–decrescendo positioning nystagmus typically provoked by lying down or turning over in the supine position. For diagnostic examination, it is elicited after a latency in the specific head position of a Dix–Hallpike maneuver (head turned 45° to one side in line with the posterior semicircular canals) or by head turns of 60–90° to the left and the right of the supine patient with the head elevated by 25° (horizontal semicircular canals) [68]. These maneuvers do not elicit attacks of VP.
